# Impacts of Cigarette Smoke (CS) on Muscle Derangement in Rodents—A Systematic Review

**DOI:** 10.3390/toxics10050262

**Published:** 2022-05-18

**Authors:** Aaron W. J. He, Shirley P. C. Ngai, Kwok Kuen Cheung, Benson W. M. Lau, Dalinda-Isabel Sánchez-Vidaña, Marco Y. C. Pang

**Affiliations:** Department of Rehabilitation Sciences, The Hong Kong Polytechnic University, Hong Kong, China; aaron.wj.he@polyu.edu.hk (A.W.J.H.); dalinda-isabel.sanchezvidana@polyu.edu.hk (D.-I.S.-V.); marco.pang@polyu.edu.hk (M.Y.C.P.)

**Keywords:** chronic obstructive pulmonary disease (COPD), cigarette smoke (CS), cross-sectional area, fiber type composition, muscle derangement, rodents

## Abstract

Cigarette smoke (CS) is the major risk factor for chronic obstructive pulmonary disease (COPD) and can induce systemic manifestations, such as skeletal muscle derangement. However, inconsistent findings of muscle derangement were reported in previous studies. The aim of the present study was to consolidate the available evidence and assess the impact of CS on muscle derangement in rodents. A comprehensive literature search of five electronic databases identified ten articles for final analysis. Results showed that the diaphragm, rectus femoris, soleus, and gastrocnemius exhibited significant oxidative to glycolytic fiber conversions upon CS exposure. In contrast, the extensor digitorum longus (EDL), plantaris, and tibialis did not exhibit a similar fiber-type conversion after CS exposure. Hindlimb muscles, including the quadriceps, soleus, gastrocnemius, and EDL, showed significant reductions in the CSA of the muscle fibers in the CS group when compared to the control group. Changes in inflammatory cytokines, exercise capacity, and functional outcomes induced by CS have also been evaluated. CS could induce a shift from oxidative fibers to glycolytic fibers in high-oxidative muscles such as the diaphragm, rectus femoris, and soleus, and cause muscle atrophy, as reflected by a reduction in the CSA of hindlimb muscles such as the quadriceps, soleus, gastrocnemius, and EDL.

## 1. Background

Cigarette smoking is a common modern addictive habit with a very negative impact on health [1,2]. Over 1.1 billion of the global population was reported to be addicted to smoking in 2015, and the associated mortality is expected to reach 8.3 million deaths by 2030 [3]. CS can induce numerous adverse effects on multiple organs leading to diseases such as chronic obstructive pulmonary disease (COPD).

COPD is a chronic progressive, not fully reversible type of airflow limitation characterized by chronic inflammation [4]. It is one of the leading causes of chronic morbidity worldwide [5,6]. The mortality of COPD currently ranks fourth globally and is expected to rank third by 2030 [3]. Previous studies showed that COPD not only induces airway inflammation directly [7], but also causes extrapulmonary manifestations such as derangement of the skeletal muscles that are involved in lower muscle strength and endurance [8,9]. For example, the quadriceps strength of people with COPD (moderate to severe level) showed a decrease of 20 to 30% compared with healthy people [10,11]. In addition, lower limb muscles (i.e., quadriceps) [12] and upper limb muscles (i.e., elbow flexors and adductor pollicis) [13,14] showed a significant reduction in mass. As the disease progresses, muscle strength was a significant predictor of mortality (RR: 0.91, 95% CI: 0.83 to 0.99, *p* = 0.036) [15]. Furthermore, a reduction of 70 cm^2^ in the cross-sectional area of the midthigh muscle, as measured by computed tomography scanning, was associated with a four-fold increase in mortality [16].

Changes in the muscle cross-sectional area (CSA) and fiber types are intrinsic mechanisms of muscle derangement [17]. In human studies, people with COPD showed a decrease in type I fibers and an increase in type II fibers in quadriceps muscles [18,19,20,21,22]. In addition to the fiber-type shift, decreases in the CSA of type I and II fibers have been described [22]. Similar results have also been found in some animal studies [23,24]. Nevertheless, inconsistent findings regarding muscle-fiber shifting and the CSA of skeletal muscles have been observed in other animal studies [9,25,26].

Hence, the aims of this systematic review were to examine the impact of CS on muscle derangement in rodents and analyze the functional consequences of muscle derangement induced by CS. The primary outcomes were changes in the proportion of muscle fiber types and their CSA, and the secondary outcomes were body mass, inflammatory markers, and functional exercise ability.

## 2. Methodology

This review was performed in accordance with preferred reporting items for systematic reviews and meta-analyses (PRISMA) guidelines.

### 2.1. Search Strategy

The detailed search strategy is listed in Table 1. Five electronic databases, including Web of Sciences (from 1970 to January 2020), Cochrane Central Register of Controlled Trials (CCRCT) (from 1999 to January 2020), Cumulative Index to Nursing and Allied Health (CIHNAL) via EBSCOhost (from 1937 to January 2020), Medline via EBSCOhost (from 1946 to Jan 2020), and EMBASE (from <1966 to January 2020), were used to search for relevant articles. Relevant articles in the references list were extracted for further screening. Two independent authors (A.W.J. He, K.K. Cheung) conducted the screening procedure independently according to the inclusion and exclusion criteria. Any disagreement was resolved by the senior author (S.P.C. Ngai).

### 2.2. Inclusion and Exclusion Criteria

Studies with a detailed description of (1) exposure to CS, (2) changes in muscle structure, such as muscle fiber type and CSA, as primary outcomes, and (3) using rodent models were included. Studies (1) using other animal models, i.e., monkey, horse, (2) adopting a review or meta-analysis, and (3) investigating outcomes not matching the preset primary outcomes were excluded.

### 2.3. Risk of Bias of Included Studies

The risk of bias in the included studies was assessed according to the criteria of the Cochrane risk of bias tool for randomized trials [27]. Five domains of risk of bias, namely, selection bias, performance bias, detection bias, attrition bias, and reporting bias, were assessed by two independent authors (A.W.J. He, K.K. Cheung) as low risk, unclear risk, high risk, and not applicable. Any disagreement was resolved by the senior author (S.P.C. Ngai).

### 2.4. Data Extraction

Data were extracted using a pre-designed data extraction form. The description of the included studies is listed in Table 2.

## 3. Results

### 3.1. Included Studies

**Figure 1:**
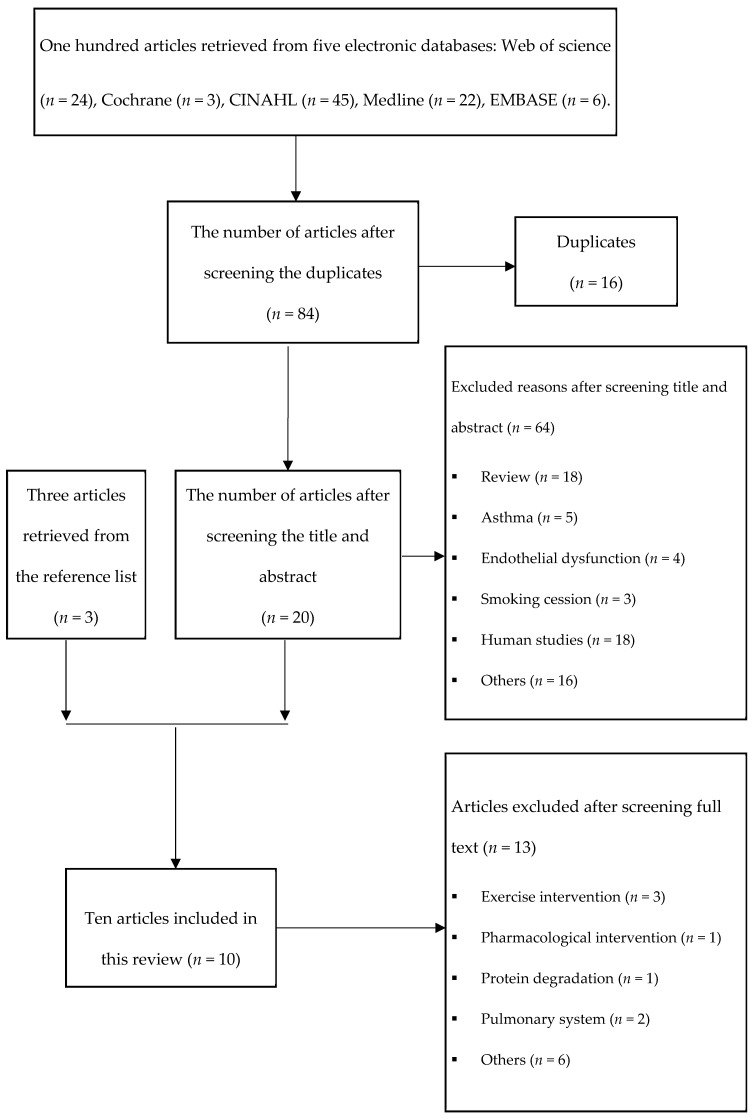
Selection procedure for articles.

A total of ten articles (*n* = 10) were included in this review with seven studies (*n* = 7) [9,23,24,25,28,29,30] retrieved from the five electronic databases and three articles (*n* = 3) [26,31,32] retrieved from reference lists. A description of the selection procedure is shown in Figure 1.

### 3.2. Characteristics of the Rodents

Three species of rodents were used in the selected studies: Hartley guinea pig, [9] mouse, [23,24,25,26,28,29,30], and Wistar-Kyoto rats [31,32]. Three strains of mouse were used: Balb/c, [24] C57Bl/6, [23,25,26,29,30], and 129/SvJ [28]. The age of the rodents ranged from 4 to 10 weeks. Nine out of ten studies [9,23,24,25,26,29,30,31,32] reported the gender of the rodents (male) whereas one study [28] did not.

### 3.3. CS Exposure Protocol

#### 3.3.1. CS Exposure System

Two types of CS exposure systems were adopted by the ten studies. Nine studies using a whole-body exposure system whereas a nose-only exposure system was chosen by one study (Table 2) [29].

#### 3.3.2. Experiment Duration

The duration of the experiments ranged from 8 to 32 weeks. An 8-week CS exposure model was adopted by 2 studies [31,32], 5 studies [9,24,26,28,29] adopted a CS exposure model for 24 weeks, and the remaining 3 studies adopted a CS exposure model for 16 [30], 18 [25], and 32 weeks (Table 2) [23].

#### 3.3.3. Frequency and Duration of CS Exposure

The frequency of CS exposure was 5 days per week in 9 studies [9,23,24,25,26,28,29,31,32] and 6 days per week in 1 study [30]. The exposure duration was reported in 6 studies [9,23,24,28,31,32] and ranged from 20 min to 24 h (7 ± 9 h per day). The remaining 4 studies [25,26,29,30] reported the number of exposures per day without stating the total exposure duration (Table 2).

#### 3.3.4. Concentration of CS Exposure

The concentration of CS exposure was described using different parameters, including the number of cigarettes per week (109 ± 39 cigarettes/week) [9,26,30,31,32], particle density in the chamber (116.5 ± 28.6 mg/m^3^) [23,28,29], carbon monoxide in the chamber (550 ± 229 ppm) [24,28], carboxyhemoglobin (HbCO) in serum (8.3%) [25], and the number puffs/min (15 puffs/min) (Table 2) [31,32].

### 3.4. Risk of Bias in the Included Studies

#### 3.4.1. Selection Bias

Random sequence generation in all of the included studies [9,23,24,25,26,28,29,30,31,32] was marked as high risk because they did not describe the method of randomization (i.e., random number table, or stratified or block randomization for low risk) (Table 3).

For allocation concealment, nine studies [9,24,25,26,28,29,30,31,32] were marked as unclear risk (Table 3) because they did not describe the approach (such as envelopes), and one study [23] had high risk of bias because the authors mentioned the term “random” without a detailed description.

#### 3.4.2. Performance Bias

Blinding of subjects was not applicable to all ten studies [9,23,24,25,26,28,29,30,31,32] because rodents as subjects cannot be blinded. Blinding of researchers was rated as unclear risk for all ten studies [9,23,24,25,26,28,29,30,31,32] because insufficient information was provided (Table 3).

#### 3.4.3. Detection Bias

Self-reported outcomes were not applicable for all ten studies [9,23,24,25,26,28,29,30,31,32] since they were animal studies. Objective outcomes had a low risk of bias for all ten studies [9,23,24,25,26,28,29,30,31,32] (Table 3) because all outcomes were assessed objectively, e.g., biopsy, immunohistology, and Western blot.

#### 3.4.4. Attrition Bias (Drop-Out)

Nearly all studies reported no animal death [9,23,24,25,26,28,30,31,32] and therefore were rated as low risk for the category of attrition bias. One study [29] reported a mortality rate of 19%, which was beyond 10%. Thus, the attrition bias of this study was rated as high risk (Table 3).

#### 3.4.5. Reporting Bias

All studies [9,23,24,25,26,28,29,30,31,32] were rated as unclear risk due to the absence of a published study protocol or protocol registration (Table 3).

### 3.5. Primary Outcomes

Six different skeletal muscle groups were examined, including the quadriceps [24], rectus femoris [25], gastrocnemius [9,23,25,26], soleus [23,26,29,30,32], EDL [29,30], tibialis [26], plantaris [26], and respiratory muscles such as the diaphragm [9] (Table 4).

#### 3.5.1. The Proportion of Muscle Fiber Types

##### Respiratory Muscle—Diaphragm

Barreiro and coworkers (2010) examined the composition of the diaphragmatic muscle [9] in guinea pigs but not in the other two species included.

When compared with the SA group, a higher proportion of type II muscle fiber was found in the CS group, but no significant difference was observed regarding type I muscle fiber after 12 weeks (SA vs. CS, type I, 37 ± 5% vs. 34 ± 2%; type II, 63 ± 5% vs. 66 ± 2%) and 16 weeks (SA vs. CS, type I, 29 ± 3% vs. 30 ± 4%; type II, 71 ± 3% vs. 70 ± 4%). However, CS exposure for 24 weeks led to a significant reduction in the oxidative fiber type I with a significant increase in the glycolytic fiber II (Table 4).

##### Lower Limb Muscle—Rectus Femoris

Only one of the included studies [23] examined the proportion of muscle fibers in the rectus femoris of mice. No between-group differences were observed for type I and type II muscle fibers after CS exposure for 8 weeks and 16 weeks. A significantly lower proportion of type I muscle fibers was observed in the CS group after 24 and 32 weeks of exposure (no original data), and a significantly higher proportion of type II muscle fibers (no original data) was observed in the same group after 24 weeks of exposure (Table 4).

##### Lower Limb Muscle—Soleus

Five out of the ten studies examined the muscle fiber proportion in the soleus of C57Bl/6 mice [23,26,29,30] and Wistar-Kyoto rats [32].

The muscle fiber type distribution in the soleus did not show any significant differences between the CS and SA groups after CS exposure for 8 weeks in C57Bl/6 mice [24] and Wistar-Kyoto rats [32]. However, another study reported a significant reduction of 19% in type II_a_ muscle fiber (fast-oxidative fiber) and an increase of 383% in type II_b/x_ muscle fiber (fast-glycolytic fiber) after 8 weeks CS exposure, with similar results after 16 weeks [30]. Other studies reported no significant difference between the groups after CS exposure for 12 and 16 weeks [23,29]. Longer periods of CS exposure produced a significant reduction in soleus type I muscle fiber and a significant increase in type II muscle fiber showed a significantly increased in the CS group as compared to the SA group after CS exposure for 24 and 32 weeks [23]. In other studies, type II_a_ muscle fiber showed a significant decrease [26] and type II_b/x_ muscle fiber showed a significant increase [29] in the CS group after CS exposure for 24 weeks.

##### Lower Limb Muscle—Gastrocnemius

Four out of ten studies evaluated muscle fiber proportions in the gastrocnemius of guinea pigs [9] and C57Bl/6 mice [23,25,26].

In guinea pigs, no differences in type I and type II muscle fibers in gastrocnemius were observed between groups at all exposure time points (Table 4).

Similar results were observed in C57Bl/6 mice after CS exposure for 8 weeks and 16 weeks [23]. In contrast, a significantly lower proportion of type I fibers and higher proportion of type II fibers were observed after CS exposure for 18 [25], 24, and 32 weeks [23].

##### Lower Limb Muscle—Other Muscles

Three out of ten studies included assessed the muscle fiber proportion in the EDL of C57Bl/6 mice [29,30] and Wistar-Kyoto rats [31]. One out of ten studies [26] examined the muscle fiber proportion in the plantaris and tibialis of C57Bl/6 mice.

The muscle fiber distribution in the EDL, plantaris, and tibialis did not show any significant difference between the CS and SA groups at any CS exposure timepoints for C57Bl/6 mice (Table 4). In Wistar-Kyoto rats, the muscle fiber distribution in the soleus and EDL did not show any significant differences between the CS and SA groups after 8 weeks of CS exposure.

#### 3.5.2. Muscle CSA

##### Respiratory Muscle—Diaphragm

Barreiro and colleagues (2010) examined the cross-sectional area (CSA) of the diaphragmatic muscle [9] in guinea pigs.

There was an increasing trend in the CSA of the diaphragm with CS exposure for 12 to 24 weeks in the CS group when compared with the SA group. The CSA of type I muscle fiber in the diaphragm showed a reduction of 9% after CS exposure for 12 weeks, then, an increase of 9 and 7% after CS exposure for 18 and 24 weeks, respectively. In contrast, the CSA of the type II muscle fibers showed a reduction of 13%, 3%, and 10% after CS exposure for 12, 16, and 24 weeks, respectively.

##### Lower Limb Muscle—Quadriceps

One out of ten studies examined the CSA of the quadriceps after CS exposure for 24 weeks in Balb/c mice [24].

The total CSA of the quadriceps showed a reduction of 15% in the CS group when compared with the SA group after CS exposure for 24 weeks (Table 5).

##### Lower Limb Muscle—Soleus

Three out of ten studies examined the CSA of the soleus in C57Bl/6 mice [23,29] and Wistar-Kyoto rats [32].

In C57Bl/6 mice, the total CSA of muscle fiber in the soleus showed no significant difference between the CS and SA group after CS exposure for 8 to 24 weeks [23,29]. On the other hand, the CSA of type I and II muscle fiber in the soleus showed a significant reduction in the CS group after CS exposure for 32 weeks [23].

In Wistar-Kyoto rats, no significant difference between the SA and CS group was found after CS exposure for 8 weeks [32] (Table 5).

##### Lower Limb Muscle—Gastrocnemius

Four out of ten studies examined the CSA of type I and II muscle fibers in the gastrocnemius of guinea pig [9], C57Bl/6 mice [23,25], and 129/SvJ mice [28].

In guinea pig, there was no significant difference between the SA and the CS group for the CSA of the gastrocnemius at any CS exposure timepoint. The CSA of type I muscle fiber reduced by 13 and 1% in the CS group after CS exposure for 12 weeks and 16 weeks, respectively. After CS exposure for 24 weeks, the CSA of type I muscle fiber showed an increase of 28% in the CS group compared with the SA group [9]. There was a similar trend in the CSA of type II muscle fiber in the gastrocnemius. The CSA of type II muscle fiber showed a reduction of 25 and 2% in the CS group after CS exposure for 12 weeks and 16 weeks, respectively, followed by an increase of 28% after 24 weeks [9] (Table 5).

In C57Bl/6 mice, the CSA of type I and II (II_a_, II_b_, and II_x_) muscle fiber in the gastrocnemius did not show any significant difference between the CS and the SA group at any timepoint [23,25].

In 129/SvJ mice, the total CSA of the gastrocnemius was significantly reduced by 12% in the CS group compared to the SA group after CS exposure for 24 weeks [28] (Table 5).

##### Lower Limb Muscle—EDL

Two out of ten studies examined the CSA of the EDL after CS exposure in C57Bl/6 mice [29] and Wistar-Kyoto rats [31].

In C57Bl/6 mice, no significant difference in the CSA of the EDL was found between the SA and the CS group after CS exposure at all timepoints [29] (Table 5).

In Wistar-Kyoto rats, the CSA of muscle type II_a_ fibers in the deep region of the EDL showed a significant reduction in the CS group where compared to the SA group after CS exposure for 8 weeks, whereas the other muscle fiber types (I, II_b_) did not show any significant differences [31] (Table 5).

### 3.6. Secondary Outcomes

#### 3.6.1. Nutritional Status

Body weight and muscle weight were used to assess nutritional status. Weight loss was detected in the CS group in all studies as a reduction in whole body mass by 3 to 23% [23,24,25,28,30,31,32] or a reduction in weight gain ratio by 30 to 78% [9,26,29] (Table 6).

#### 3.6.2. Inflammatory Markers

One study investigated the levels of inflammatory markers in the diaphragm and gastrocnemius after CS exposure [9].

Tumor Necrosis Factor-α (TNF-α), Interleukin-6 (IL-6), and total number of inflammatory cells were adopted as inflammatory markers.

No statistically significant difference was found for the levels of TNF-α and IL-6 in the gastrocnemius and diaphragm after CS exposure at all timepoints.

The concentration of TNF-α in the gastrocnemius in the CS group showed no change after CS exposure for 12 weeks, but there was a reduction of 17% after CS exposure for 16 weeks, and an increase of 14% after CS exposure for 24 weeks. The concentration of TNF-α in the diaphragm increased by 31% and 52% after CS exposure for 12 and 16 weeks, respectively, then reduced by 15% after CS exposure for 24 weeks.

The concentration of IL-6 in the gastrocnemius in the CS group increased by 17% after CS exposure for 12 weeks, reduced by 6% after CS exposure for 16 weeks, and increased by 27% after CS exposure for 24 weeks. The concentration of IL-6 in the diaphragm showed an increase of 20% after CS exposure for 12 weeks, an increase of 42% after CS exposure for 16 weeks, and a reduction of 3% after CS exposure for 24 weeks.

Furthermore, no statistically significant difference was observed in the number of inflammatory cells, including leukocytes and macrophages, in the gastrocnemius and diaphragm after CS exposure at all timepoints. The number of inflammatory cells in the gastrocnemius in the CS group showed an increase of 39% after CS exposure for 12 weeks, an increase of 50% after CS exposure for 16 weeks, and an increase of 77% after CS exposure for 24 weeks. The number of inflammatory cells in the diaphragm showed an increase of 46% after CS exposure for 12 weeks, 67% after CS exposure for 16 weeks, and 48% after CS exposure for 24 weeks when compared with the SA group.

#### 3.6.3. Functional Outcomes

Three studies examined exercise capacity and functional performance using a treadmill test [23,28,30]. The parameters evaluated included maximum speed (V_max_) (m/min), time to reach exhaustion (min), maximal oxygen uptake (mL/min/kg) (VO_2 max_), running time (min), and run distance (m). No significant difference in V_max_ was found in the CS group [30]. Furthermore, a small (8%) but insignificant difference in the time to reach exhaustion was found after CS exposure for 8 weeks, whereas a significant reduction of 21% was found in the CS group after 16 weeks [30]. A significant difference in the VO_2 max_ was found after CS exposure for 16 weeks in the CS group [23]. Furthermore, a significant reduction of 10% in running time was found in the CS group compared to the SA group [28]. A significant reduction of 13% in running distance was observed for the CS group in the same study [28] and a second study also reported a significant reduction [23].

## 4. Discussion

### 4.1. Effects of Exposure to CS on the Percentage of Muscle Fiber Types in Skeletal Muscles

**Diaphragm**

Results indicated that long-term CS exposure for at least 24 weeks could cause a shift from oxidative to glycolytic type muscle fiber in the diaphragm.

**Rectus femoris**

The rectus femoris also showed a high percentage of oxidative muscle fibers (type I and II_a_) [33].

**Soleus**

The present study found that the proportion of oxidative muscle fiber (type I or II_a_) in the soleus showed a significant reduction, and the proportion of glycolytic muscle fiber (type II or II_b/x_) was significantly increased in the CS group when compared with the SA group, particularly after CS exposure for 24 weeks. On the other hand, there were inconsistent results in the soleus after CS exposure for 8, 12, and 16 weeks.

**Gastrocnemius, EDL, Plantaris and Tibialis**

In gastrocnemius, the proportion of type I muscle fiber was increased, and the proportion of type II muscle fiber was reduced after CS exposure for 32 weeks, whereas there were inconsistent results for each type of muscle fiber after CS exposure for 8 to 24 weeks.

The EDL [34], plantaris [34,35], and tibialis [34] have a high percentage of glycolytic muscle fibers (type II_b_ and II_x_) in rodents, which increases the tolerance to a CS-induced hypoxic environment. The lack of a change in the muscle fiber type in the gastrocnemius agrees with the compensatory changes in muscle fiber type in the glycolytic muscles after CS exposure [34].

**Explanations for inconsistent findings in the percentage of muscle fiber types**

**Soleus**

Inconsistent results may have been caused by the following reasons. First, the studies used different species of rodents such as Wistar-Kyoto rats and C57Bl/6 mice. The sensitivity and response to CS exposure in rats and mouse were different. Also, there was no standard parameter to evaluate the intensity of CS exposure, even when using the same exposure timepoint. Second, the absence of a standard parameter to evaluate the intensity of CS exposure could have contributed to the inconsistent results. In other words, the lack of a standardize measurement to evaluate the intensity of CS exposure makes it difficult to compare the results from different studies. Third, the use of different CS exposure systems could have accounted for the discrepant results. For example, the nose-only system directly affected the pulmonary system whereas the whole-body exposure system could have had a bigger impact than the nose-only system. This may be another reason for no significant difference in type II_a_ and II_b/x_ fibers in the soleus between the SA and CS groups after CS exposure for 12 weeks.

**Gastrocnemius**

The inconsistent results could be due to the use of different species of rodent including guinea pig and C57Bl/6 mouse, and the absence of a standard parameter to evaluate the intensity of CS exposure.

**Mechanism of effects of CS on the percentage of muscle fiber types in skeletal muscles**

The skeletal muscles are composed of four types of muscle fibers, namely, the slow oxidative type I, fast oxidative type II_a_, fast intermediate type II_x_, and fast glycolytic type II_b_. The diaphragm [35] has a high percentage of oxidative muscle fibers whose major components include type I and II_a_.

The duration of exposure and concentration of toxins are known to be essential factors that are associated with the incidence and severity of disease. However, it was difficult to compare data between studies due to non-standardized protocols. However, data from the same studies may provide some clues.

Krüger et al. (2015) [23] investigated the effect of CS exposure duration (at a fixed dose of TPM = 140 mg/m^3^) for 8, 16, 24, and 32 weeks on mouse muscles. Despite a non-linear time-dependent relationship, significant differences in muscle fiber type redistribution from oxidative to glycolytic type in the diaphragm, rectus femoris, and soleus were observed from 24 weeks onwards, clearly indicating a potential effect on muscle fiber distribution when CS exposure duration and cumulative concentration are increased. On the other hand, Nakatani and co-workers found no significant differences in the soleus [32] and EDL [31] of Wistar-Kyoto rats when different daily exposure doses were used (i.e., low-dosage with 23 cigarettes/day, medium-dosage with 26 cigarettes/day, and high-dosage with 30 cigarettes/ day) for 8 weeks, except that the CSA of the deep region of the EDL in the high CS dosage was significantly smaller [31]. Although it may appear that the effect of concentration was less apparent when compared to time, it is worth noting that the sensitivity to cigarette exposure may be different between species or even between strains. In fact, our recent study [36] has clearly demonstrated a dose-dependent effect on fiber-type shifting. It is believed that both time and dose play critical roles in modulating fiber type derangement, and further investigation of these two relationships is warranted.

CS contains three major toxic substances—carbon monoxide (CO), nicotine, and tar—and they play an important role in stimulating the transformation of muscle fiber types.

CO reduces oxygen transport and release [37] because it has a 210-fold greater affinity than O_2_ for hemoglobin (Hb) [37]. As a result, HbCO increases and oxygen-hemoglobin is reduced. The displacement of O_2_ by CO from hemoglobin leads to decreasing amounts of O_2_ in the blood, which creates a hypoxic environment.

Nicotine, a selective alpha-1 adrenoreceptor blocker, reduces the blood supply by irritating the capillary contraction that is caused by its inhalation [38]. A reduction in blood supply also contributes to the hypoxic environment. Tar has been found to impair mitochondrial respiratory chain function, leading to less oxygenation [39]. To adapt to the hypoxic condition and improve oxygenation, there is a compensatory shift from oxidative muscle fibers (type I and II_a_) to glycolytic fibers (type II_b/x_). Therefore, oxidative muscle fibers are more susceptible to CS exposure.

### 4.2. Effects of Exposure to CS on the CSA of Muscle Fiber Types in Skeletal Muscles

The CSA of the hindlimb muscles including the quadriceps, rectus femoris, soleus, gastrocnemius, and EDL showed a statistically significant reduction in type I and II muscle fibers in the CS group when compared to the SA group, whereas the percentage of oxidative muscle fibers and the CSA of oxidative muscle fibers were decreased.

**Mechanism of effects of CS on the CSA of muscle fiber types in skeletal muscles**

An increase in protein degradation is commonly observed in various muscle-atrophic conditions, such as immobilization, denervation, unloading, dexamethasone treatment, and IL-1-induced cachexia [40]. The primary pathway contributing to the increase in protein degradation is the ATP-dependent, ubiquitin-proteasome pathway [40]. Muscle RING finger-1 (MuRF-1) and muscle atrophy F-box (MAFbx) are the specific E3 ubiquitous ligases involved in the regulation of muscle atrophy [40] and are often used as biomarkers for muscle atrophy. In addition to protein degradation, a reduction in protein synthesis can also lead to a reduced cross-sectional area as a result of muscle atrophy. An increase in the expression of myostatin in muscle, which is known as a negative regulator of muscle growth, can lead to inactivation of protein kinase (Akt) (a trigger for protein synthesis) [41], and ultimately, suppression of cultured myoblast proliferation [42]. In vivo studies have further indicated that myostatin inhibited satellite cell proliferation and differentiation [43,44].

In the present study, muscle atrophy, as indicated by a reduction in CSA, in muscles like the quadriceps, soleus, gastrocnemius, and EDL in animals occurred upon cigarette smoking. These changes in limb muscles suggested muscle atrophy as a result of either protein degradation and/or suppressed protein synthesis. In human studies, quadriceps muscle biopsies obtained from smokers showed a decrease in protein synthesis (a significant decrease of 59.46% in fractional synthesis rate) as well as a concomitant increase in protein degradation (a significant increase of 45% in the expression of MAFbx) when compared to those obtained from non-smokers [45]. In an animal study, mRNA levels of MAFBx and MuRF1 were increased in the gastrocnemius of mice upon exposure to CS for 40 days [30]. Additionally, in vitro studies [44,46] also illustrated an increase in MAFBx and MuRF1 in atrophied myotubes cells when exposed to CS. Caron and colleagues (2013) [47] identified a significant reduction in the phosphorylated Akt/total Akt ratio (*p* = 0.02) in the gastrocnemius of mice exposed to CS for 24 weeks.

Apoptosis of muscle satellite cells may also play a role in muscle atrophy. In the present review, two studies examined the effects of CS on the apoptosis of muscles. Krüger et al. (2015) [23] found that CS upregulated of genes in cell death (e.g., Fas) and Ma et al. (2017) [24] detected that CS downregulated the serum response factor (SRF) target genes that regulate the development and differentiation of skeletal muscle. These findings are in line with other studies [48,49] in which Pax7+/Nes+ satellite cells were significant lower in soleus fibers exposed to CS (*p* < 0.01).

Altogether, these studies demonstrate that cigarette exposure is capable of impairing the process of protein synthesis and enhancing protein degradation in both humans and animals. While the effects of the active ingredients in cigarette like nicotine and tar on skeletal muscle are not completely understood, perhaps a more simple and straightforward explanation is that chronic cigarette smoking simply induces a hypoxic microenvironment. In fact, Basic et al. (2012) [28] have clearly demonstrated that skeletal muscles of mice exposed to chronic cigarette smoke showed reduced exercised tolerance, reduced CSA, as well as significantly enhanced expression of VHL, PHD2, UBE2D1, all of which are elements of the ubiquitination cascade. In addition, the authors have also shown upregulation of HIF-1α mRNA, and upregulation of HIF-1 and VEGF protein in muscles, indicating hypoxia and impaired muscle capillarization. It is therefore clear that cigarette smoke-generated hypoxia may account for, at least in part, reductions in the CSA of skeletal muscles.

The reduction of the CSA of oxidative muscle fibers when adapting to the hypoxic environment occurred prior to the shift from oxidative to glycolytic fiber types after CS exposure. The CSA of oxidative type II_a_ muscle fiber in the deep region of the EDL was reduced after a short exposure to CS (8 weeks) in rats, and as the duration of exposure to CS continued, the CSA of the oxidative and glycolytic fibers reduced.

Upon hypoxia, oxygen uptake by the locomotor muscles was impaired, and so was their functional capacity. In the treadmill test, a reduction of 8 to 21% in the time to reach exhaustion, a reduction of 10% in running time, and a reduction of 13% in running distance were found after CS exposure. In addition, a statistically significant difference between the SA and CS group for VO_2max_ was found after CS exposure for 16 weeks. Furthermore, an impairment of the contractile properties (20% decrease in force generated from a force-frequency curve at 80 Hz, *p* = 0.087) [29] and fatigue resistance (reduction of 43%) [48] of the muscles were also found after CS exposure.

Inflammatory cytokines (such as TNF-α) are also involved in muscle atrophy after exposure to CS through destabilizing MyoD (a major trigger participating in the proliferation and differentiation of satellite cells) and inducing apoptosis of satellite cells, leading to the imbalance of protein synthesis and protein degradation (a decrease in protein synthesis or/and an increase in protein degradation) [50]. Moreover, De Paepe et al. (2008) [25] found that when TNF-α receptor-2 knockout mice were exposed to CS, muscle atrophy was attenuated in comparison with wild-type mice.

### 4.3. Effects of Exposure to CS on Body Mass

Compared to the control group, the CS group showed a lower body mass by 3 to 23% [23,24,28,30,31,32] or a reduced weight gain ratio by 30 to 78% [9,26,29]. Such phenomena could be associated with a catabolic and anabolic imbalance within skeletal muscle, or an altered neurological regulation of food intake and energy expenditure [51]. Caron and colleagues [47] on one hand reported that CS exposure upregulated catabolic signaling targets such as MuRF1, Atrogin-1, and FoxO3 in the gastrocnemius. On the other hand, they also observed reduced phosphorylation of Akt and expression of glycogen synthase kinase 3-β. Such an imbalance between protein degradation and protein synthesis could explain the reduced muscle size, and thus, reasonably influence overall body mass.

From a neurological perspective, previous studies showed that nicotine was associated with an increased level of dopamine and serotonin, thereby inhibiting food ingestion [52] and suppressing appetite leading to weight loss [52,53]. In addition, nicotine stimulated and activated proopiomelanocortin (POMC) neurons (in the hypothalamus) to release melanocortin peptides such as β-endorphin and α-melanocyte stimulating hormone, both of which act on the melanocortin receptor to suppress food intake [53]. Other studies have reported that nicotine mediates fatty tissue metabolism [52,53] and increases levels of leptin, a satiety hormone that binds to hypothalamic receptors to promote the release of anorexigenic neurotransmitters [52,53], to contribute further to the loss of body mass.

## 5. Limitation and Recommendation

### 5.1. Limitations of the Studies Involved

As there was a lack of a standardized assessment form to evaluate the risk bias of the animal studies, the risk bias of studies involved could not be evaluated.

### 5.2. Recommendation

As discussed, there was no standard protocol for the animal model with COPD induced by CS. The variation in CS exposure systems (i.e., nose-only exposure system or whole-body exposure system), exposure duration of CS, daily and/or cumulative concentration of exposure may lead to different extents of skeletal muscle derangement. Therefore, a standard protocol taking into consideration all of these variables is needed in future research.

## 6. Conclusions

CS could induce a muscle fiber shift from oxidative fibers (type I and II_a_) to glycolytic fibers (type II_b/x_) in high-oxidative muscles such as the diaphragm, rectus femoris, and soleus. In addition, CS induced muscle atrophy, as reflected by a reduction in the CSA of hindlimb muscles such as the quadriceps, rectus femoris, soleus, gastrocnemius, and EDL. In line with this muscle derangement, exercise ability was attenuated when analyzing the maximum speed, time to exhaustion, maximal oxygen uptake, running time, and running distance in a treadmill test.

## Figures and Tables

**Table 1:** Search strategy and keywords.

**ID**	**Chronic Obstructive Pulmonary Disease**
1.	COPD
2.	COAD
3.	COBD
4.	Chronic Airflow Obstruction
5.	Airflow Obstruction, Chronic
6.	Chronic Obstructive Airway Disease
7.	Chronic Obstructive Pulmonary Disease
8.	Chronic Obstructive Lung Disease
9.	Pulmonary Disease, Chronic Obstructive
10.	Lung Diseases, Obstructive
11.	Emphysema*
12.	(Obstruct* and (pulmonary or lung or airway* or airflow* or bronchitis* or respirat*))
13.	1 or 2 or 3 or 4 or 5 or 6 or 7 or 8 or 9 or 10 or 11 or 12
**ID**	**Muscle Derangement**
14.	Muscle Derangement
15.	Muscle Dysfunction
16.	Muscle Weakness
17.	14 OR 15 OR 16
**ID**	**Cigarette Smoking**
18.	Cigar Smoking
19.	Smoking, Cigar
20.	Tobacco Smoking
21.	Smoking, Tobacco
22.	Cigarette Smoking
23.	Smoking, Cigarette
24.	18 OR 19 OR 20 OR 21 OR 22 OR 23
**ID**	**Animal**
25.	No human
26.	Animal
27.	25 OR 26
28.	13 AND 17 AND 24 AND 27

Note: * is truncation symbol. This is used to truncate a word in order to search for different forms of the same word.

**Table 2:** Studies included and CS exposure protocol.

Author (Year)	Rodents			Cigarette Smoke (CS) Exposure Protocol					
	Species	Strain	Age (Weeks)	Exposure Period (Weeks)	Sample Size		Type of CS Exposure	No. of Cigarettes/Week	The Concentration of CS Exposure
					SA (n)	CS (n)			
Ma (2018) [24]	Mouse	Balb/c	6–8	24	15	15	Whole body	45	▪ The conc. of CO = 500–800 ppm
Basic (2012) [28]	Mouse	129/SvJ	8–10	24	8	6	Whole body	-	▪ The conc. TPM = 100 mg/m^3^ ▪ The conc. of CO = 350 ppm
Rinaldi (2012) [29]	Mouse	C57Bl/6	8	12	6	6	Nose-only	40	▪ The conc. of TPM = 149.5 mg/m^3^
				24	12	12			
Tang (2010) [30]	Mouse	C57Bl/6	8	8	6	6	Whole body	60	▪ No original data provided
				16	6	6			
Barreiro (2010) [9]	Guinea pig	Hartley	4	12	7	7	Whole body	35	▪ No original data provided
				16	7	7			
				24	7	7			
Gosker (2009) [26]	Mouse	C57Bl/6	7–9	24	13	13	Whole body	100	▪ No original data provided
De Paepe (2008) [25]	Mouse	C57Bl/6	8	18	4	4	Whole body	100	▪ The conc. of HbCO = 8.3%
Krüger (2015) [23]	Mouse	C57Bl/6	6–8	8	10	10	Whole body	-	▪ TPM = 140 mg/m^3^
				16	10	10			
				24	10	10			
				32	10	10			
Nakatani (2003) [31]	Rat	Wistar-Kyoto	10	L: 8	20	20	Whole body	115	▪ Rate of smoke delivering: 15 puffs/min
				M: 8	20	20		130	
				H: 8	20	20		150	
Nakatani (2002) [32]	Rat	Wistar-Kyoto	10	L: 8	20	20	Whole body	115	▪ Rate of smoke delivering: 15 puffs/min
				M: 8	20	20		130	
				H: 8	20	20		150	

Note: CO = carbon monoxide, conc. = concentration, CS = cigarette smoke group, H = high-dose, HbCO = carboxyhemoglobin, L = low-dose, M = medium-dose, SA = sham air group, TPM = total particular matter.

**Table 3:** Risk of bias in the included studies.

Author (Year)	Selection Bias		Performance Bias	Detection Bias	Attrition Bias	Reporting Bias
	Random Sequence Generation	Allocation Concealment	Blinding of Researcher	Blinding of Outcome Assessment: Objective Measures	Incomplete Outcome Data: All Outcomes	Selective Reporting
Ma (2018) [24]	High risk	Unclear risk	Unclear risk	Low risk	Low risk	Unclear risk
Basic (2012) [28]	High risk	Unclear risk	Unclear risk	Low risk	Low risk	Unclear risk
Rinaldi (2012) [29]	High risk	Unclear risk	Unclear risk	Low risk	High risk	Unclear risk
Tang (2010) [30]	High risk	Unclear risk	Unclear risk	Low risk	Low risk	Unclear risk
Barreiro (2010) [9]	High risk	Unclear risk	Unclear risk	Low risk	Low risk	Unclear risk
Gosker (2009) [26]	High risk	Unclear risk	Unclear risk	Low risk	Low risk	Unclear risk
De Paepe (2008) [25]	High risk	Unclear risk	Unclear risk	Low risk	Low risk	Unclear risk
Krüger (2015) [23]	High risk	High risk	Unclear risk	Low risk	Low risk	Unclear risk
Nakatani (2003) [31]	High risk	Unclear risk	Unclear risk	Low risk	Low risk	Unclear risk
Nakatani (2002) [32]	High risk	Unclear risk	Unclear risk	Low risk	Low risk	Unclear risk

Note: Random sequence generation was ranked high risk because the studies did not describe the methods of randomization (i.e., random number table); allocation concealment was ranked unclear risk because the studies did not mention the methods (i.e., envelopes) or high risk because the studies only mentioned random; blinding of researchers was ranked unclear risk because studies did not describe whether two different people conducted the assessments and treatments; bias of objective measures was low risk because outcomes were assessed in an objective manner (i.e., biopsy); attrition bias was ranked low risk because when no rats died in the studies, and high risk when the death rate was >10%; selective reporting was ranked unclear risk because no protocol was published in the studies.

**Table 4:** Percentage of muscle fiber types at different time points during CS exposure.

Muscles	Species /Strain	Author (Year)	Muscle Fiber Type	CS Exposure Period (Weeks)	Groups		Summary
					SA	CS	
Diaphragm	Guinea pigs	Barreiro (2010) [9]	Type I (%)	12	37 ± 5	34 ± 2	▪ In guinea pigs, the percentage of type I in CS group was lower than SA group after CS exposure from 12 to 24 weeks. ▪ The percentage of muscle fiber type I in CS group was statistically significantly lower than SA group (*p* < 0.05) after CS exposure for 24 weeks.
				16	29 ± 3	30 ± 4	
				24	35 ± 5	30 ± 2 *	
			Type II (%)	12	63 ± 5	66 ± 2	▪ In guinea pigs, the percentage of type II fibers in CS group was higher than SA group after CS exposure from 12 and 24 weeks. ▪ The percentage of muscle fiber type II fibers in CS group was statistically significantly higher than SA group (*p* < 0.05) after CS exposure for 24 weeks.
				16	71 ± 3	70 ± 4	
				24	65 ± 5	70 ± 2 *	
Rectus femoris	Mouse, C57Bl/6	Krüger (2015) [23]	Type I (%)	8	No significant difference		▪ In C57Bl/6 mouse, the percentage of type I fibers in CS group was statistically significantly lower compared with SA group after CS exposure for 24 and 32 weeks.
				16	No significant difference		
				24	↓* CS vs. SA		
				32	↓* CS vs. SA		
			Type II (%)	8	No significant difference		▪ In C57Bl/6 mouse, the percentage of type II fibers in CS group was statistically significantly higher compared with SA group after CS exposure for 24 and 32 weeks.
				16	No significant difference		
				24	↑* CS vs. SA		
				32	↑* CS vs. SA		
Soleus	Mouse, C57Bl/6	Krüger (2015) [23]	Type I (%)	8	No significant difference		▪ In C57Bl/6 mouse, the percentage of type I fibers in CS group was decreased compared with SA group after CS exposure for 24 and 32 weeks.
		Tang (2010) [30]			No significant difference		
		Rinaldi (2012) [29]		12	No significant difference		
		Krüger (2015) [23]		16	No significant difference		
		Tang (2010) [30]		18	No significant difference		
		Krüger (2015) [23]		24	↓* CS vs. SA		
		Rinaldi (2012) [29]		24	No significant difference		
		Gosker (2009) [26]		24	No significant difference		
		Krüger (2015) [23]		32	↓* CS vs. SA		
	Wistar-Kyoto rats	Nakatani (2002) [32]		8	No significant difference		▪ In Wistar-Kyoto rats, the mean difference in percentage of type I fibers between SA and CS groups was not statistically significant after CS exposure for 8 weeks.
	Mouse, C57Bl/6	Krüger (2015) [23]	Type II (%)	8	No significant difference		▪ In C57Bl/6 mouse, the percentage of type II fibers in CS group was increased compared with SA group after CS exposure for 24 and 32 weeks.
				16	No significant difference		
				24	↑* CS vs. SA		
				32	↑* CS vs. SA		
	Wistar-Kyoto rats	Nakatani (2002) [32]		8	No significant difference		▪ In Wistar-Kyoto rats, the mean difference in percentage of type II fibers between SA and CS group was not statistically significant after CS exposure for 8 weeks.
	Mouse, C57Bl/6	Tang (2010) [30]	Type II_a_ (%)	8	↓* CS vs. SA		▪ In C57Bl/6 mouse, the percentage of type II_a_ fibers in CS group was decreased compared with SA group after CS exposure for 8, 18, and 24 weeks.
		Rinaldi (2012) [29]		12	No significant difference		
		Tang (2010) [30]		18	54.4 ± 7.3	44.0 ± 8.0 *	
		Rinaldi (2012) [29]		24	No significant difference		
		Gosker (2009) [26]			↓* CS vs. SA		
		Tang (2010) [30]	Type II_b_ (%)	8	0.53 ± 0.37	2.56 ± 1.34 *	▪ In C57Bl/6 mouse, the percentage of type II_b_ fibers in CS group was statistically significantly higher than SA group after CS exposure for 8 and 18 weeks.
				18	0.53 ± 0.37	12.9 ± 3.8 *	
		Rinaldi (2012) [29]	Type II_b/x_ (%)	12	No significant difference		▪ In C57Bl/6 mouse, the percentage of type II_b/x_ fibers in CS group was statistically significantly higher than SA group after CS exposure for 24 weeks.
		Gosker (2009) [26]		24	No significant difference		
		Rinaldi (2012) [29]		24	9.8 ± 0.7	15.1 ± 1.2 *	
Gastrocnemius	Guinea pigs	Barreiro (2010) [9]	Type I (%)	12	10 ± 3	10 ± 4	▪ In guinea pig, the percentage of type I fibers in CS group was higher than SA group after CS exposure for 12, 16, and 24 weeks.
				16	11 ± 5	8 ± 3	
				24	9 ± 3	13 ± 4	
	Mouse, C57Bl/6	Krüger (2015) [23]		8	No significant difference		▪ In C57Bl/6 mouse, the percentage of type I fibers in CS group was statistically significantly lower than SA group after CS exposure for 24 and 32 weeks.
		Krüger (2015) [23]		16	No significant difference		
		Gosker (2009) [26]		24	No significant difference		
		Krüger (2015) [23]		24	↓* CS vs. SA		
		Krüger (2015) [23]		32	↓* CS vs. SA		
	Guinea pigs	Barreiro (2010) [9]	Type II (%)	12	90 ± 3	90 ± 4	▪ In guinea pig, the percentage of type II fibers in CS group was lower than SA group after CS exposure for 12, 16, and 24 weeks.
				16	89 ± 5	92 ± 3	
				24	91 ± 3	87 ± 6	
	Mouse, C57Bl/6	Krüger (2015) [23]		8	No significant difference		▪ In C57Bl/6 mouse, the percentage of type II fibers in CS group was statistically significantly higher than SA group after CS exposure for 24 and 32 weeks.
				16	No significant difference		
				24	↑* CS vs. SA		
				32	↑* CS vs. SA		
		De Paepe (2008) [25]	Type II_a_ (%)	18	37.2 ± 5.7	20.9 ± 2.8 *	▪ In C57Bl/6 mouse, the percentage of type II_a_ fibers in CS group was statistically significantly lower than SA group after CS exposure for 18 weeks.
		Gosker (2009) [26]		24	No significant difference		
		De Paepe (2008) [25]	Type II_b_ (%)	18	44.7 ± 4.7	76.5 ± 3.5 *	▪ In C57Bl/6 mouse, the percentage of type II_b_ fibers in CS group was statistically significantly higher than in SA group after CS exposure for 18 weeks.
		Gosker (2009) [26]		24	No significant difference		
		De Paepe (2008) [25]	Type II_x_ (%)	18	12.4 ± 2.4	14.5 ± 3.8	▪ In C57Bl/6 mouse, the percentage of type II_b/x_ fibers in CS group was lower than SA group after CS exposure for 18 weeks.
		Gosker (2009) [26]		24	No significant difference		
EDL	Mouse, C57Bl/6	Tang (2010) [30]	Type I (%)	8	No significant difference		▪ In C57Bl/6 mouse, there was no statistically significant difference in the percentage of type I fibers between SA and CS groups after CS exposure from 8 to 24 weeks.
		Rinaldi (2012) [29]		12			
		Tang (2010) [30]		18			
		Rinaldi (2012) [29]		24			
	Wistar-Kyoto rats	Nakatani (2003) [31]		8	No significant difference		▪ In Wistar-Kyoto rats, there was no statistically significant difference in the percentage of type I fibers between SA and CS groups after CS exposure for 8 weeks.
	Mouse, C57Bl/6	Tang (2010) [30]	Type II_a_ (%)	8	14.3 ± 6.1	14.3 ± 6.1	▪ In C57Bl/6 mouse, there was no statistically significant difference in the percentage of type II_a_ fibers between SA and CS groups after CS exposure for 8 to 18 weeks.
		Rinaldi (2012) [29]		12	No significant difference		
		Tang (2010) [30]		18	14.3 ± 6.1	14.3 ± 6.1	
		Rinaldi (2012) [29]		24	No significant difference		
	Wistar-Kyoto rats	Nakatani (2003) [31]		8	No significant difference		▪ In Wistar-Kyoto rats, there was no statistically significant difference in the percentage of type II_a_ fibers between SA and CS groups after CS exposure for 8 weeks.
	Mouse, C57Bl/6	Tang (2010) [30]	Type II_b_ (%)	8	83.6 ± 5.5	83.6 ± 5.5	▪ In C57Bl/6 mouse, there was no statistically significant difference in the percentage of type II_b_ fibers between SA and CS groups after CS exposure from 8 to 18 weeks.
				18	83.6 ± 5.5	83.6 ± 5.5	
	Rats, Wistar-Kyoto	Nakatani (2003) [31]		8	No significant difference		▪ In Wistar-Kyoto rats, there was no statistically significant mean difference in the percentage of type II_b_ fibers between SA and CS groups after CS exposure for 8 weeks.
	Mouse, C57Bl/6	Tang (2010) [30]	Type II_b/x_ (%)	8	No significant difference		▪ In C57Bl/6 mouse, there was no statistically significant difference in the percentage of type II_b/x_ fibers between SA and CS groups after CS exposure from 8 to 24 weeks.
		Rinaldi (2012) [29]		12			
		Tang (2010) [30]		18			
		Rinaldi (2012) [29]		24			
Plantaris	Mouse, C57Bl/6	Gosker (2009) [26]	Type I (%)	24	No significant difference		▪ In C57Bl/6 mouse, there was no statistically significant difference in the percentage of type II_a_ fibers between SA and CS groups after CS exposure for 24 weeks.
			Type II_a_ (%)				▪ In C57Bl/6 mouse, there was no statistically significant difference in the percentage of type II_a_ fibers between SA and CS groups after CS exposure for 24 weeks.
			Type II_b/x_ (%)				▪ In C57Bl/6 mouse, there was no statistically significant difference in the percentage of type II_b/x_ fibers between SA and CS groups after CS exposure for 24 weeks.
Tibialis	Mouse, C57Bl/6	Gosker (2009) [26]	Type I (%)	24	No significant difference		▪ In C57Bl/6 mouse, there was no statistically significant difference in the percentage of type II_a_ fibers between SA and CS groups after CS exposure for 24 weeks.
			Type II_a_ (%)				▪ In C57Bl/6 mouse, there was no statistically significant difference in the percentage of type II_a_ fibers between SA and CS groups after CS exposure for 24 weeks.
			Type II_b/x_ (%)				▪ In C57Bl/6 mouse, there was no statistically significant difference in the percentage of type II_b/x_ fibers between SA and CS groups after CS exposure for 24 weeks.

Note: Data is presented as the mean ± SD. EDL = extensor digitorum longus, CS = cigarette smoke, SA = sham air group, * = statistically significant mean difference between SA and CS group, ↓* CS vs. SA = statistically significant decrease in CS group compared to SA group, ↑* CS vs. SA = statistically significant increase in CS group compared to SA group.

**Table 5:** Cross-sectional area (CSA) of muscle fibers at different time points during CS exposure.

Muscles	Species /Strain	Author (Year)	Muscle Fiber Type	CS Exposure Period (Weeks)	Groups		Summary
					SA	CS	
Diaphragm	Guinea pigs	Barreiro (2010) [9]	Type I (µm^2^)	12	734 ± 143	666 ± 304	▪ In guinea pig, the CSA of type I in CS group was larger than in SA group after CS exposure for 12, 16, and 24weeks by −68 µm^2^, 54 µm^2^, 60 µm^2^, respectively. ▪ The mean difference in CSA of type I between SA and CS groups was not statistically significantly after CS exposure
				16	593 ± 146	647 ± 195	
				24	697 ± 192	757 ± 134	
			Type II (µm^2^)	12	850 ± 135	743 ± 127	▪ In guinea pig, the CSA of type II fibers in CS group was lower than SA group after CS exposure for 12, 16, and 24 weeks by −107 µm^2^, −58 µm^2^, −105 µm^2^, respectively. ▪ The mean difference for the CSA of type I fibers between SA and CS groups was not statistically significant after CS exposure.
				16	743 ± 127	685 ± 148	
				24	1013 ± 130	908 ± 203	
Quadriceps	Mouse, Balb/c	Ma (2018) [24]	Total CSA (µm^2^)	24	48.43 ± 1.17	41.05 ± 0.10 *	▪ In Balb/c mouse, the total CSA of muscle fibers was significantly decreased by 7.4 µm^2^ in CS group compared with SA group after CS exposure for 24 weeks.
Rectus femoris	Mouse, C57Bl/6	Krüger (2015) [23]	Type I (µm^2^)	8	No significant difference		▪ In C57Bl/6 mouse, the CSA of type I fibers in CS group was statistically significantly decreased compared with SA group after CS exposure for 24 weeks.
				16	No significant difference		
				24	↓* CS vs. SA		
			Type II (µm^2^)	8	No significant difference		▪ In C57Bl/6 mouse, the CSA of type II fibers in CS group was statistically significantly decreased compared with SA group after CS exposure for 24 weeks.
				16	No significant difference		
				24	↓* CS vs. SA		
Soleus	Rats, Wistar-Kyoto	Nakatani (2002) [32]	Total CSA (µm^2^)	8	No significant difference		▪ In Wistar-Kyoto rats, no statistically significant difference in the total CSA of muscle fibers between SA and CS groups was observed after CS exposure for 8 weeks.
	Mouse, C57Bl/6	Krüger (2015) [23]	Type I (µm^2^)	8	No significant difference		▪ In C57Bl/6 mouse, the CSA of type I fibers in CS group was statistically significantly decreased compared with SA group after CS exposure for 32 weeks.
				16	No significant difference		
				24	No significant difference		
				32	↓* CS vs. SA		
			Type II (µm^2^)	8	No significant difference		▪ In C57Bl/6 mouse, the CSA of type II fibers in CS group showed a statistically significantly decrease when compared with SA group after CS exposure for 32 weeks.
				16	No significant difference		
				24	No significant difference		
				32	↓* CS vs. SA		
Gastrocnemius	Mice, 129/SvJ	Basic (2012) [28]	Total CSA (µm^2^)	24	2771.16	2429.3 *	▪ In 129/SvJ mouse, the total CSA of muscle fibers in CS group was statistically significantly lower compared with SA group after CS exposure for 24 weeks.
	Guinea pigs	Barreiro (2010) [9]	Type I (µm^2^)	12	894 ± 256	779 ± 198	▪ In guinea pig, the CSA of type I fibers in CS group was greater than SA group after CS exposure for 12, 16, and 24 weeks by −115 µm^2^, −10 µm^2^, 286 µm^2^, respectively. ▪ The mean difference of CSA of type I fibers between SA and CS groups showed no statistically significant difference after CS exposure.
				16	797 ± 200	787 ± 212	
				24	1010 ± 399	1296 ± 582	
	Mouse, C57Bl/6	Krüger (2015) [23]		8	No significant difference		▪ In C57Bl/6 mouse, the CSA of type I fibers in CS group was lower when compared with SA group after CS exposure for 18 weeks. ▪ In CS group, CSA was statistically significantly lower than SA group after CS exposure for 32 weeks.
				16	No significant difference		
		De Paepe (2008) [25]		18	550 ± 190	510 ± 130	
		Krüger (2015) [23]		24	No significant difference		
				32	↓* CS vs. SA		
	Guinea pigs	Barreiro (2010) [9]	Type II (µm^2^)	12	1154 ± 325	1129 ± 247	▪ In guinea pig, the CSA of type II fibers in CS group was consistently lower than SA group after CS exposure for 12, 16, and 24 weeks by −25 µm^2^, −10 µm^2^, −217 µm^2^, respectively. ▪ The mean difference of the CSA of type I fibers between SA and CS groups was not statistically significantly different after CS exposure.
				16	1148 ± 228	1125 ± 246	
				24	1545 ± 523	1328 ± 248	
	Mouse, C57Bl/6	Krüger (2015) [23]		8	No significant difference		▪ In C57Bl/6 mouse, the CSA of type II fibers in CS group was statistically significantly lower than SA group after CS exposure for 32 weeks.
				16	No significant difference		
				24	No significant difference		
				32	↓* CS vs. SA		
		De Paepe (2008) [25]	Type II_a_ (µm^2^)	18	510 ± 110	600 ± 200	▪ In C57Bl/6 mouse, the CSA of type II_a_ fibers in CS group was greater by 90 µm^2^ compared with SA group after CS exposure for 18 weeks.
			Type II_b_ (µm^2^)	18	980 ± 170	1300 ± 320	▪ In C57Bl/6 mouse, the CSA of type II_b_ fibers in CS group was greater by 320 µm^2^ compared with SA group after CS exposure for 18 weeks.
			Type II_x_ (µm^2^)	18	680 ± 210	690 ± 200	▪ In C57Bl/6 mouse, the CSA of type II_x_ fibers in CS group was greater by 10 µm^2^ compared with SA group after CS exposure for 18 weeks.
EDL	Mouse, C57Bl/6	Rinaldi (2012) [29]	Total CSA (µm^2^)	12	No significant difference		▪ In C57Bl/6 mouse, the difference in the total CSA was not significant between SA and CS groups after CS exposure for 12 and 24 weeks.
				24	No significant difference		
EDL(Superficial region)	Rats, Wistar-Kyoto	Nakatani (2003) [31]	Type I (µm^2^)	8	No significant difference		▪ In Wistar-Kyoto rats, the difference in the CSA of type I fibers was not significant between SA and CS groups after CS exposure for 8 weeks.
			Type II (µm^2^)	8	No significant difference		▪ In Wistar-Kyoto rats, the difference in the CSA of type II fibers was not significant between SA and CS groups after CS exposure for 8 weeks.
EDL (Deep region)			Type II_a_ (µm^2^)	8	↓* CS vs. SA		▪ In Wistar-Kyoto rats, the CSA of type II_a_ fibers in CS group was statistically significantly lower than SA group after CS exposure for 8 weeks.
			Type II_b_ (µm^2^)	8	No significant difference		▪ In Wistar-Kyoto rats, the difference of the CSA of type II_b_ was no significant between SA and CS groups after CS exposure for 8 weeks.

Note: Data is presented as the mean ± SEM. EDL = extensor digitorum longus, CS = cigarette smoke, SA = sham air group, * = statistically significant mean difference between SA and CS group, ↓* CS vs. SA = statistically significant decrease in CS group compared to SA group.

**Table 6:** Summary of the body weight at different time points during CS exposure.

Author (Year)	Characteristics of Rodents		CS Exposure Period (Weeks)	Groups		Summary
	Species	Strain		SA	CS	
Ma (2018) [24]	Mouse	Balb/c	24	34.5 ± 0.8	26.6 ± 0.4 *	▪ In Balb/c mouse, the body weight in CS group was statistically significantly lower than SA group by 7.9 g (*p* < 0.05) after CS exposure for 24 weeks.
Krüger (2015) [23]		C57Bl/6	8	20.9 ± 1.2	19.5 ± 0.9	▪ In C57Bl/6 mouse, the body weight in CS group was consistently lower than SA group by 1.3 to 7.9 g after CS exposure for 8 to 32 weeks. ▪ There was an inconsistent statistically significant lower body weight in CS group after CS exposure for 8 weeks, while the body weight was consistently statistically significantly lower in CS group compared with SA group after CS exposure for 16 to 32 weeks. ▪ The trend for the mean difference in body weight increased for CS exposure from 8 to 18 weeks, then the trend decreased from 18 to 24 weeks, and lastly, the trend was relatively stable from 24 to 32 weeks.
Tang (2010) [30]				34.9 ± 3.4	30.2 ± 3.2 *	
Krüger (2015) [23]			16	25.7 ± 0.9	24.3 ± 1.0 *	
Tang (2010) [30]				34.9 ± 3.4	29.3 ± 3.7 *	
De Paepe (2008) [25]			18	35.1 ± 0.4	28.9 ± 0.6 *	
Krüger (2015) [23]			24	29.1 ± 0.9	27.8 ± 1.0 *	
Krüger (2015) [23]			32	31.5 ± 1.4	30.1 ± 1.6 *	
Basic (2012) [28]		129/SvJ	24	36.9 ± 1.01	31.6 ± 1.16 *	▪ In 129/SvJ mouse, the body weight in CS group was statistically significantly lower than SA group by 5.3 g after CS exposure for 24 weeks.
Nakatani (2003) [31]	Rats	Wistar-Kyoto	8	L: 359 ± 15	348 ± 22	▪ In Wistar-Kyoto rats, the body weight in CS group was lower than SA group by 10 to 17 g after CS exposure for 8 weeks.
				M: 358 ± 14	348 ± 21	
				H: 360 ± 25	343 ± 11	

Note: Data is presented as the mean ± SEM. * = significant difference between SA and CS groups, CS = cigarette smoke group, H = high-dose, L = low-dose, M = medium-dose, SA = sham air group.

## Data Availability

Not applicable.
