# Correction: He et al. Impacts of Cigarette Smoke (CS) on Muscle Derangement in Rodents—A Systematic Review. *Toxics* 2022, *10*, 262

**DOI:** 10.3390/toxics12010058

**Published:** 2024-01-11

**Authors:** Aaron W. J. He, Shirley P. C. Ngai, Kwok Kuen Cheung, Benson W. M. Lau, Dalinda-Isabel Sánchez-Vidaña, Marco Y. C. Pang

**Affiliations:** Department of Rehabilitation Sciences, The Hong Kong Polytechnic University, Hong Kong, China; aaron.wj.he@polyu.edu.hk (A.W.J.H.); dalinda-isabel.sanchezvidana@polyu.edu.hk (D.-I.S.-V.); marco.pang@polyu.edu.hk (M.Y.C.P.)

## Errors in Tables

In the original publication [1], there were mistakes in Tables 2, 3, 5, 6 as published. The publication years of Ma (2017), Tang (2012), and Krüger (2005) were incorrect. The corrected reference should be Ma (2018) in Tables 2, 3, 5, 6 and Tang (2010) and Krüger (2015) in Tables 2 and 3.

In Table 2, the concentration of CS Exposure in Gosker (2009) was not mentioned, and the conc. of HbCO = 8.3% should be deleted. The reported age of 6–8 weeks and the exposure period of 16 weeks in De Paepe (2008) were incorrect. The correct age was 8 weeks, and the exposure period was 18 weeks.

In Table 4, the data of the gastrocnemius in C57Bl/6 mice missed Gosker’s (2009) data. The data of type I and types II_a_, II_b_, and II_x_ of the gastrocnemius at 24 weeks of CS exposure in Gosker (2009) should be added; there was no significant difference.

## Text Correction

There was an error in the original publication.

The in-text description of nine studies is on pages 11, 12, 14, and 15. A correction has been made to ten studies on pages 11, 12, 14, and 15. Five out of the ten studies examined muscle fiber proportions in the soleus of C57Bl/6 mice [23,26,29,30] and Wistar–Kyoto rats [32]. (p. 11, subheading: Lower Limb Muscle—Soleus, line 1); four out of the ten studies evaluated muscle fiber proportions in the gastrocnemius of guinea pigs [9] and C57Bl/6 mice [23,25,26] (p. 11, subheading: Lower Limb Muscle—Gastrocnemius, line 1); Three out of ten studies included assessed the muscle fiber proportion in the EDL of C57Bl/6 mice [29,30] and Wistar-Kyoto rats [31]. One out of ten studies [26] examined the muscle fiber proportion in the plantaris and tibialis of C57Bl/6 mice (p. 11, subheading: Lower Limb Muscle—Other Muscles, line 1); one out of the ten studies examined the CSA of the quadriceps after CS exposure for 24 weeks in Balb/c mice [24] (p. 12, subheading: Lower Limb Muscle—Quadriceps, line 1); three out of the ten studies examined the CSA of the soleus in C57Bl/6 mice [23,29] and Wistar–Kyoto rats [32] (p. 14, subheading: Lower Limb Muscle—Soleus, line 1); four out of the ten studies examined the CSA of types I and II muscle fibers in the gastrocnemius of guinea pigs [9], C57Bl/6 mice [23,25], and 129/SvJ mice [28] (p. 14, subheading: Lower Limb Muscle—Gastrocnemius, line 1); two out of the ten studies examined the CSA of the EDL after CS exposure in C57Bl/6 mice [29] and Wistar–Kyoto rats [31] (p. 15, subheading: Lower Limb Muscle—EDL, line 1). The incorrect statement in the original publication stated that “a significantly higher proportion of type I fibers and lower proportion of type II fibers were observed after CS exposure for 18 [25], 24, and 32 weeks [23]” (p. 11, subheading: Lower Limb Muscle—Gastrocnemius, line 6). However, upon further review, it has been determined that the correct observation is “a significantly lower proportion of type I fibers and higher proportion of type II fibers were observed after CS exposure for 18 [25], 24, and 32 weeks [23].” 

As Ref. 26 is added (p. 5, Section 3.3.4. Concentration of CS Exposure, line 2), the number of cigarettes per week is 109 ± 39 cigarettes per week. 

## References

The level of carboxyhemoglobin (HbCO) in serum (8.3%) is only reported in Ref. 25 but not in Ref. 26; consequently, the number of cigarettes per week adds that in Ref. 26 (p. 5, Section 3.3.4. Concentration of CS Exposure). The CSA of the gastrocnemius of C57Bl/6 mice were reported in Refs. 23 and 25, but not in Ref. 29 (p. 14, subheading: Lower Limb Muscle—Gastrocnemius, line 2). The three included studies for functional outcomes should be Refs. 23, 28, and 30, but not Ref. 26 (p. 16, Section 3.6.3. Functional Outcomes). Ref. 25 should be added for the reduction in whole body mass (p. 15, Section: 3.6.1. Nutritional Status) and Ref. 31 should be removed from p. 11 subheading: Lower Limb Muscle—Soleus, line 5.

The authors state that the scientific conclusions are unaffected. This correction was approved by the Academic Editor. The original publication has also been updated.
